# Eye movement-related brain activity during perceptual and cognitive processing

**DOI:** 10.3389/fnsys.2014.00062

**Published:** 2014-04-24

**Authors:** Andrey R. Nikolaev, Sebastian Pannasch, Junji Ito, Artem V. Belopolsky

**Affiliations:** ^1^Research Group Experimental Psychology, KU LeuvenLeuven, Belgium; ^2^Department of Psychology, Technische Universität DresdenDresden, Germany; ^3^Research Center Juelich, Institute of Neuroscience and Medicine (INM-6)Juelich, Germany; ^4^Department of Cognitive Psychology, Vrije Universiteit AmsterdamAmsterdam, Netherlands

**Keywords:** eye movements, saccade, smooth pursuit, eye tracking, free viewing, EEG, local field potentials, fMRI

For several decades researchers have been recording electrical brain activity associated with eye movements in attempt to understand their neural mechanisms. However, recent advances in eye-tracking technology have allowed researchers to use eye movements as the means of segmenting the ongoing brain activity into episodes relevant to cognitive processes in scene perception, reading, and visual search. This opened doors to uncovering the active and dynamic neural mechanisms underlying perception, attention and memory in naturalistic conditions. The present eBook contains a representative collection of studies from various fields of visual neuroscience that use this cutting edge approach of combining eye movements and neural activity.

The majority of the articles in the eBook combine the measurement of eye movements with the recording of the electroencephalogram (EEG) in human subjects performing various psychological tasks. The most common methodological approach is examination of the EEG activity time-aligned to certain eye movement events, such as the onset of a fixation or a start of a saccadic eye movement (Fischer et al., 2013; Frey et al., 2013; Henderson et al., 2013; Hutzler et al., 2013; Nikolaev et al., 2013; Richards, 2013; Simola et al., 2013). Several works employ the time-frequency and synchrony analysis (Fischer et al., 2013; Hoffman et al., 2013; Ito et al., 2013; Nakatani and Van Leeuwen, 2013; Nakatani et al., 2013).

The advantage of simultaneous EEG and eye movement recording is most evident in investigation of perceptual and cognitive processes during free eye movement behavior. Saccades in free viewing are guided by the bottom-up and top-down attentional mechanisms. To study interactions between these mechanisms Fischer et al. (2013) explored the eye fixation-related potentials (EFRP) and EEG power during extended picture viewing. The difference between the mechanisms was reflected in the EFRP components and in the power of the frontal beta- and theta activity. Nakatani and Van Leeuwen (2013) recorded EEG and eye movements during free viewing of the Necker cube. They found that saccades and blinks facilitate perceptual switches. Moreover, the amplitude of alpha activity preceding these eye events predicted whether a blink or a saccade results in the switch. Nikolaev et al. (2013) examined the pre-saccadic EEG activity during free visual exploration of a natural scene in anticipation of a memory test. Their findings illustrate how pre-saccadic activity differentiates encoding of visual information and selection of a target for the next fixation. Simola et al. (2013) investigated attention and emotion processes by analyzing EFRPs in free viewing. They found that emotional processing depends on the overt attentional resources. Ito et al. (2013) observed interaction between low and high frequency components in the local field potentials (LFP) recorded in the visual cortex of monkeys performing voluntary saccades during natural scene viewing. They concluded that the cross-frequency interaction is a manifestation of the mechanism which coordinates oculomotor behavior and sensory processing. Hoffman et al. (2013) recorded the fixation-related neural activity in the human and macaque hippocampus during unrestricted visual search. They found in both species that the fixation-related phase alignment of the hippocampal low-frequency oscillations depends on the visual task.

Not only EEG or LFP recordings, but fMRI can be also related to eye movements in free viewing of scenes (Marsman et al., 2013). The authors investigated the neural correlates of ambient and focal processing using fixation-based event-related (FIBER) fMRI in combination with independent component analysis. They reported the eye-movement related activity in the ventromedial and ventrolateral visual cortices.

As shown in this eBook, reading studies also benefit from the combination of EEG and eye movement recordings. Henderson et al. (2013) devised an advanced procedure to correct EFRP for eye movement artifacts. After correction the early EFRP components were different between reading and pseudo-reading (control) condition. To investigate parafoveal pre-processing in reading Hutzler et al. (2013) applied the X-mask in the task of new/old word judgment. The time course of the EFRP indicated dynamical interference of the parafoveal mask with foveal word recognition. Frey et al. (2013) studied decision making during reading using EFRP. They compared EFRP when participants fixated the words related and unrelated to the predefined decision conditions. The late EFRP components appeared to be indicative of the semantic decision.

Furthermore, two studies investigated brain activity during performance of particular saccade tasks. Nakatani et al. (2013) measured cross-frequency phase coupling in peri-fixation brain activity during semantic judgment in the controlled saccade conditions. They concluded that the cross-frequency phase synchrony constitutes a plausible mechanism for tagging of fixation information. Richards (2013) used prosaccade and antisaccade tasks to examine the cortical sources of the related EEG activity. He demonstrated the value of this approach to differentiate the brain activities associated with general preparatory processes and with eye movement execution.

The methodological aspects of co-registration of brain activity with eye movements are addressed in the work which introduces a video projection system for high-speed, gaze-contingent stimulus presentation (Richlan et al., 2013).

Besides the chapters which describe the brain activity related to saccadic eye movements, the eBook also features a review on cognitive processes involved in smooth pursuit eye movements in humans and animals (Fukushima et al., 2013). The authors discuss involvement of cognitive processes in the smooth pursuit including neural substrates, behavioral evidence and clinical correlations.

Taken together, the present eBook demonstrates that relating brain activity and eye movements is a fruitful way of studying a wide range of psychological processes without imposing an artificial task structure. This approach is particularly useful to demonstrate how brain dynamics underlying perceptual and cognitive processes unfolds over time in naturalistic conditions.

## Conflict of interest statement

The authors declare that the research was conducted in the absence of any commercial or financial relationships that could be construed as a potential conflict of interest.
